# Supplementary material for: Comparative multiomics analysis of cell physiological state after culture in a basket bioreactor
Source: Sci Rep. 2022 Nov 23;12:20161. doi: 10.1038/s41598-022-24687-4 (PMC9686226; doi:10.1038/s41598-022-24687-4)
Supplement: Supplementary file 1 — Supplementary Information 1. [file 41598_2022_24687_MOESM1_ESM.zip › raw data/Transcriptomic raw data/7.AS/2.dasplot/TE1vsTC1/SE/2_RPS24_NW_023666048.1_13824999_13825109_-@NW_023666048.1_13822091_13822112_-@NW_023666048.1_13821620_13821708_-.pdf]

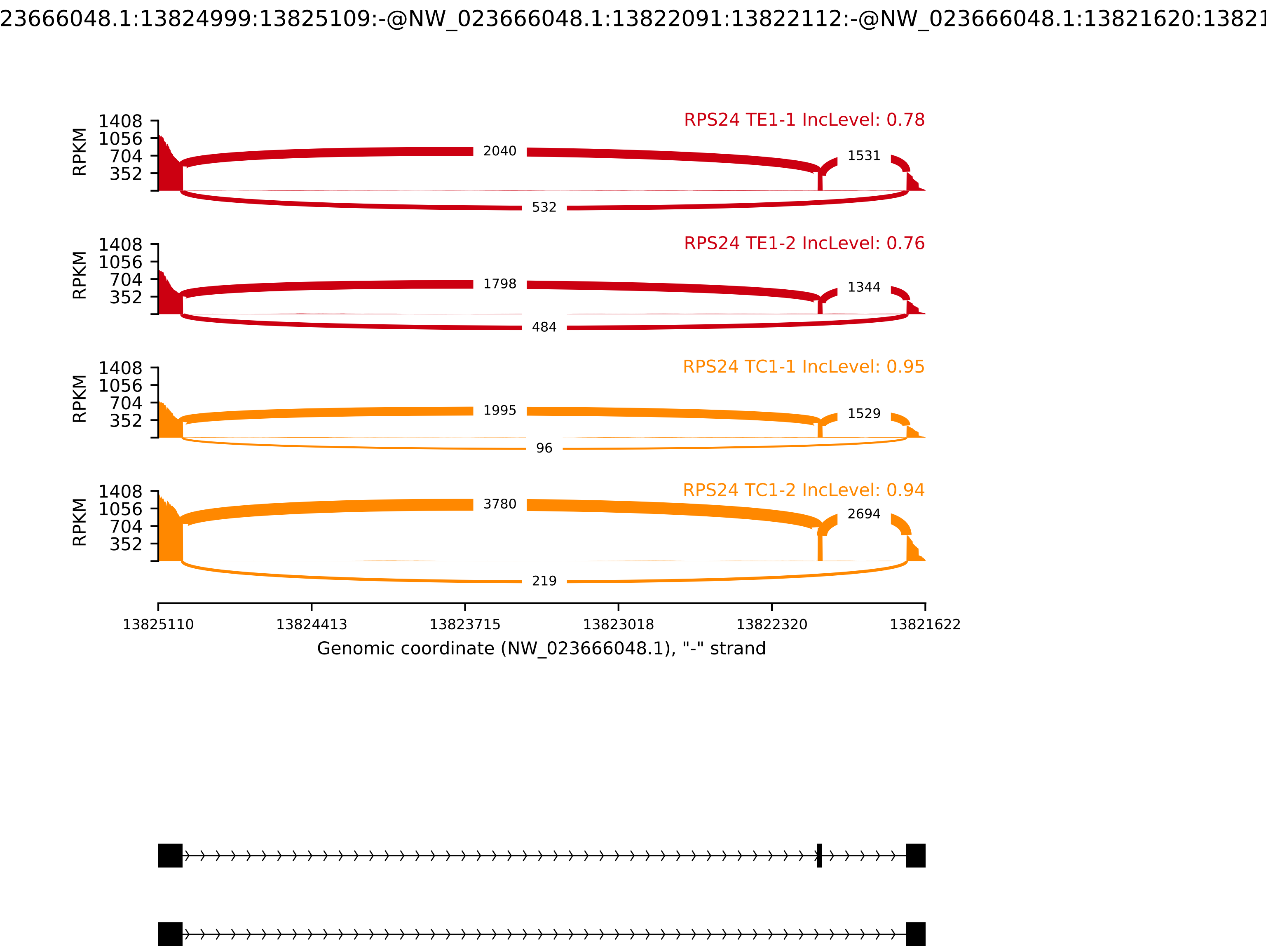

23666048.1:13824999:13825109:-@NW_023666048.1:13822091:13822112:-@NW_023666048.1:13821620:1382
RPKM
1408
1056
704
352
RPS24 TE1-1 IncLevel: 0.78
2040
1531
532
RPS24 TE1-2 IncLevel: 0.76
1798
1344
484
RPS24 TC1-1 IncLevel: 0.95
1995
1529
96
RPS24 TC1-2 IncLevel: 0.94
3780
2694
219
13825110
13824413
13823715
13823018
13822320
13821622
Genomic coordinate (NW_023666048.1), "-" strand
